# Extensor Mechanism Disruption after Total Knee Arthroplasty: A Case Series and Review of Literature

**DOI:** 10.7759/cureus.479

**Published:** 2016-02-04

**Authors:** Raju Vaishya, Amit Kumar Agarwal, Vipul Vijay

**Affiliations:** 1 Orthopaedics, Indraprastha Apollo Hospitals

**Keywords:** extensor mechanism disruption, total knee arthroplasty, quadriceps tendon rupture

## Abstract

Extensor mechanism disruption following total knee arthroplasty (TKA) is a rare but devastating complication. These patients may require revision of the implants, but even then, it may not be possible to restore the normal function of the knee after the disruption. The patterns of extensor mechanism disruption can broadly be classified into three types: suprapatellar (quadriceps tendon rupture), transpatellar (patellar fracture), or infrapatellar (patellar tendon rupture). Infrapatellar tendon ruptures are the worst injuries, as they carry maximum morbidity and are challenging to manage. The disruption of the extensor mechanism may occur either intra-operatively or in the immediate postoperative period due to an injury. The treatment of extensor mechanism complications after TKA may include either nonsurgical management or surgical intervention in the form of primary repair or reconstruction with autogenous, allogeneic, or synthetic substitutes. We have provided an algorithm for the management of extensor mechanism disruption after TKA.

## Introduction

Extensor mechanism disruption is one of the reported reasons for revision surgery after total knee arthroplasty (TKA) with the incidence of 0.17 - 1.4% [1]. It is also a frequent source of postoperative morbidity. The extensor mechanism includes the quadriceps muscle and tendon, medial and lateral retinaculum, patellofemoral and patellotibial ligaments, patella, patellar tendon, and tibial tubercle. Discontinuity in any of these components can lead to an extensor mechanism disruption and render an otherwise perfectly good TKA useless. Extensor mechanism-related complications can occur either intraoperatively or postoperatively. Commonly reported causes during the surgery include avulsion or tendon injury during the exposure, improper patellar resection, and damage to the blood supply due to injudicious lateral retinacular release or multiple prior surgeries. The postoperative causes may include tissue necrosis arising from infection, component malalignment, and trauma. Various treatment options are available for managing these challenging problems, and recent advances in alternative reconstruction techniques have yielded promising results [2]. We are presenting our experience of 10 knees (nine cases) with extensor mechanism disruption following TKA.

## Materials and methods

In a retrospective study, we present a series of nine cases (with 10 knees) of extensor mechanism rupture associated with TKA. All the patients who had primary TKA during 2000-2014 by the senior author were reviewed. Informed patient consent was obtained at the time of treatment. Out of 5,254 patients, nine were found to have extensor mechanism disruption following TKA. All these primary TKAs were done using a midline anterior (modified Insall’s) approach, using a posterior stabilized cemented prosthesis, Scorpio^TM ^  Single Axis Total Knee System (Stryker, Kalamazoo, MI). 

## Results

**Figure 1:**
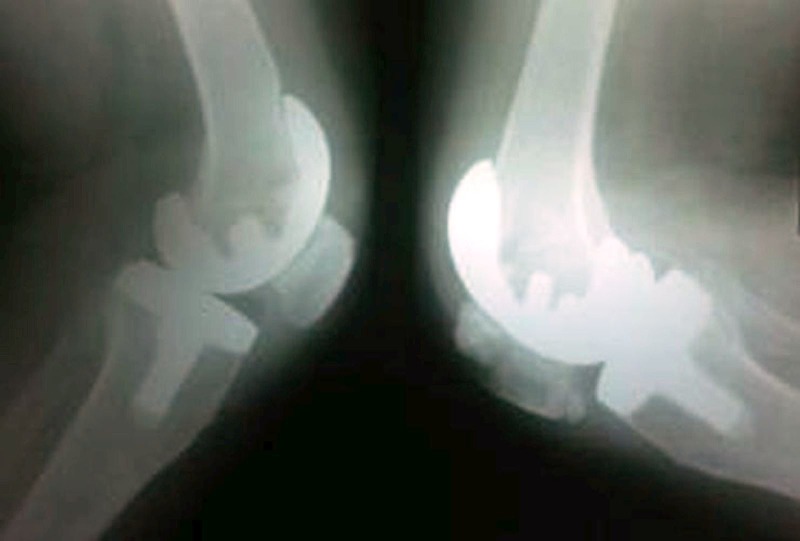
Lateral radiograph of a patient with a left patellar fracture three months after total knee arthroplasty

Complications involving the extensor mechanism occur in 1% to 12% of patients following primary TKA. The average age of the patient was 59.6 years (range: 32 - 72 years). There were seven females and two males. One patient had an injury to both the knees. Four patients had rheumatoid arthritis (RA) and five had osteoarthritis (OA) as their primary diagnosis. All the patients were operated using a midline medial parapatellar approach, except one with a severe valgus deformity who was exposed by a lateral parapatellar approach. The average duration of injury since primary surgery was 8.16 weeks (range: 1 - 24 weeks). Most patients have had trivial domestic injuries due to falling on stairs or a floor, injuries due to forceful bending of the knees during exercise, or while sitting down on the toilet. One patient with RA had severe knee stiffness preoperatively with a range of motion (ROM) of 10-20 degrees. This patient sustained a patellar fracture three months postoperatively due to forceful bending of the knee while doing exercises (Figure 1).

**Figure 2:**
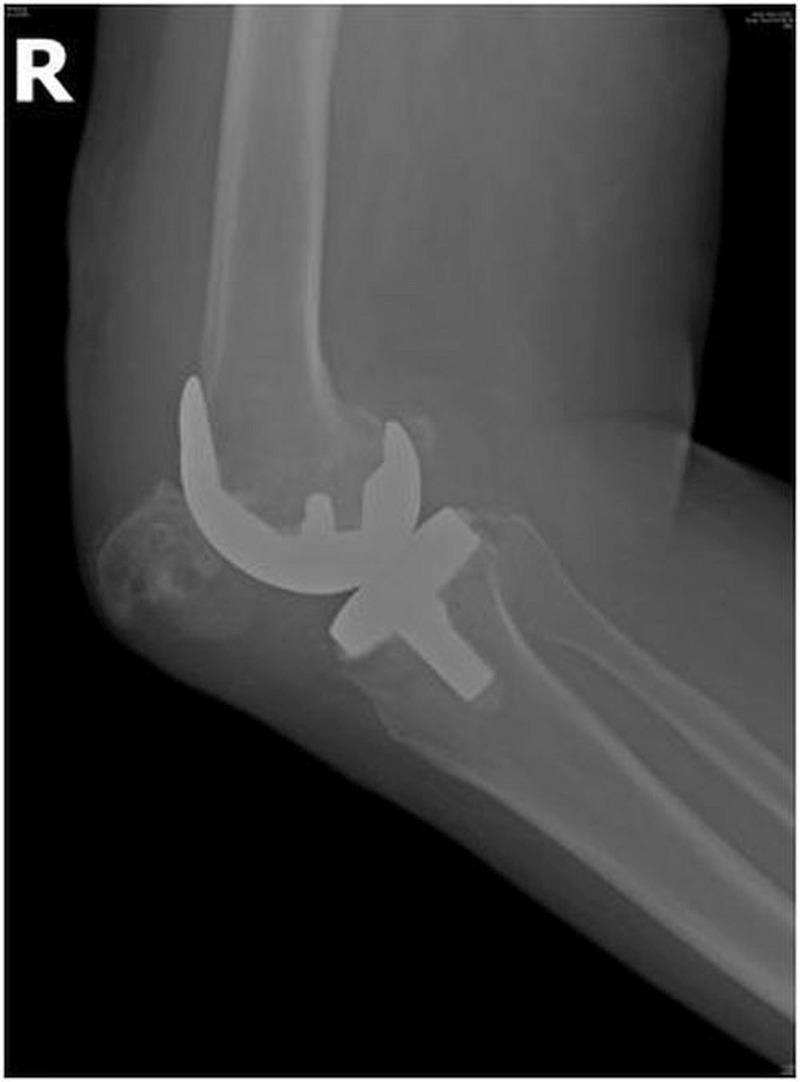
Lateral radiograph of a patient showing lateral patellar dislocation on the right side after TKA.

**Figure 3:**
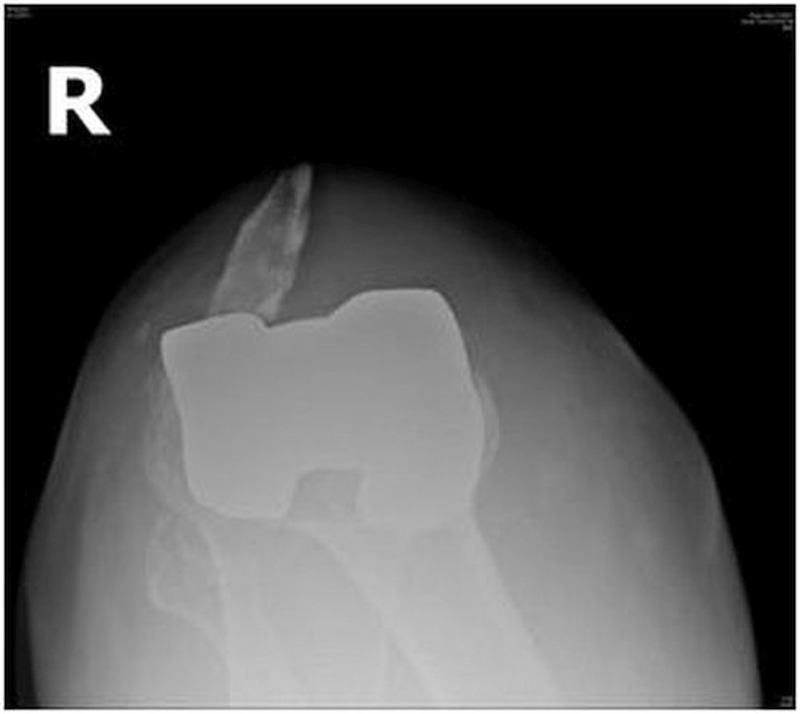
Merchant view of a patient showing a lateral patellar dislocation on the right side after TKA.

**Figure 4:**
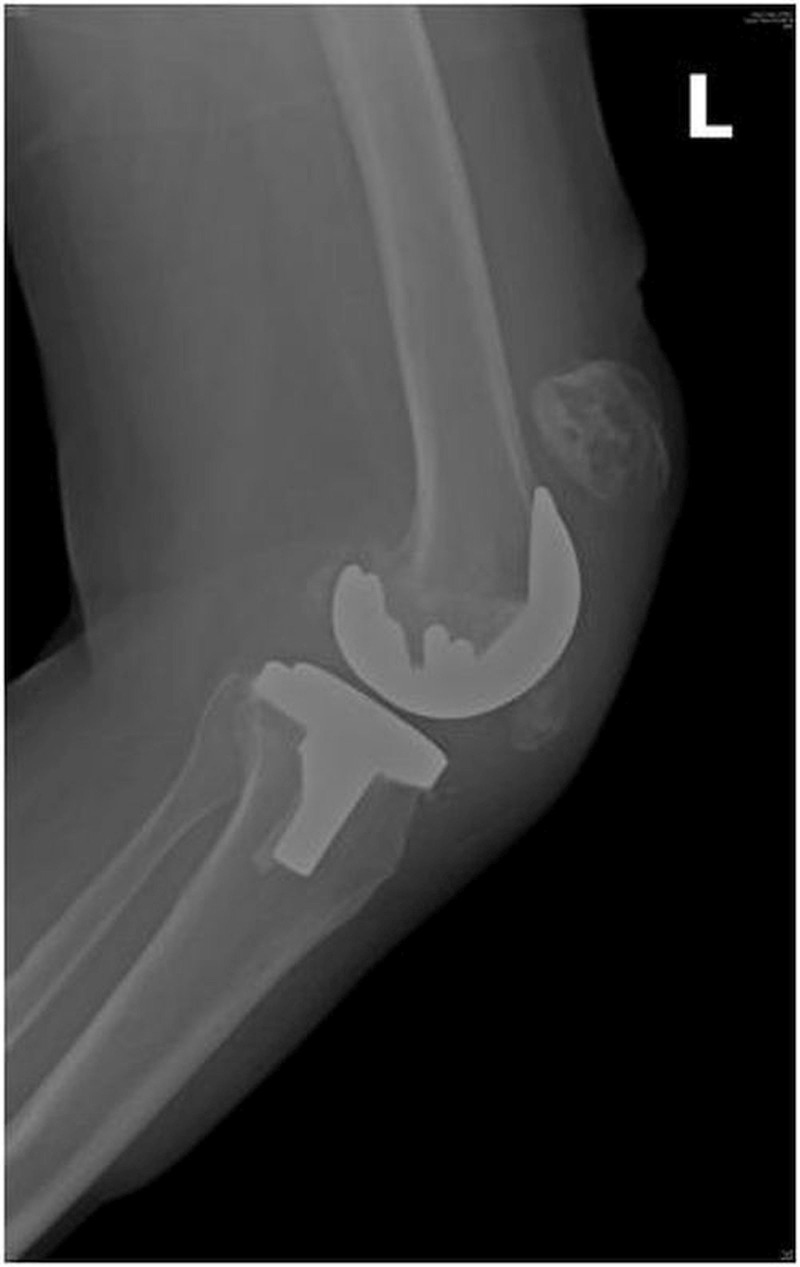
Lateral radiograph of a patient showing a patellar fracture on the left side after TKA

One patient with OA sustained an intraoperative injury to the patella while everting the patella with a towel clip that led to the crushing of osteoporotic bone and subsequent fracture within one week of surgery. One patient with bilateral TKA incurred injuries to both the knees following a fall from some stairs and sustained a lateral patellar dislocation on one side (Figures 2-3) and a patellar fracture on the other side (Figure 4).

**Figure 5:**
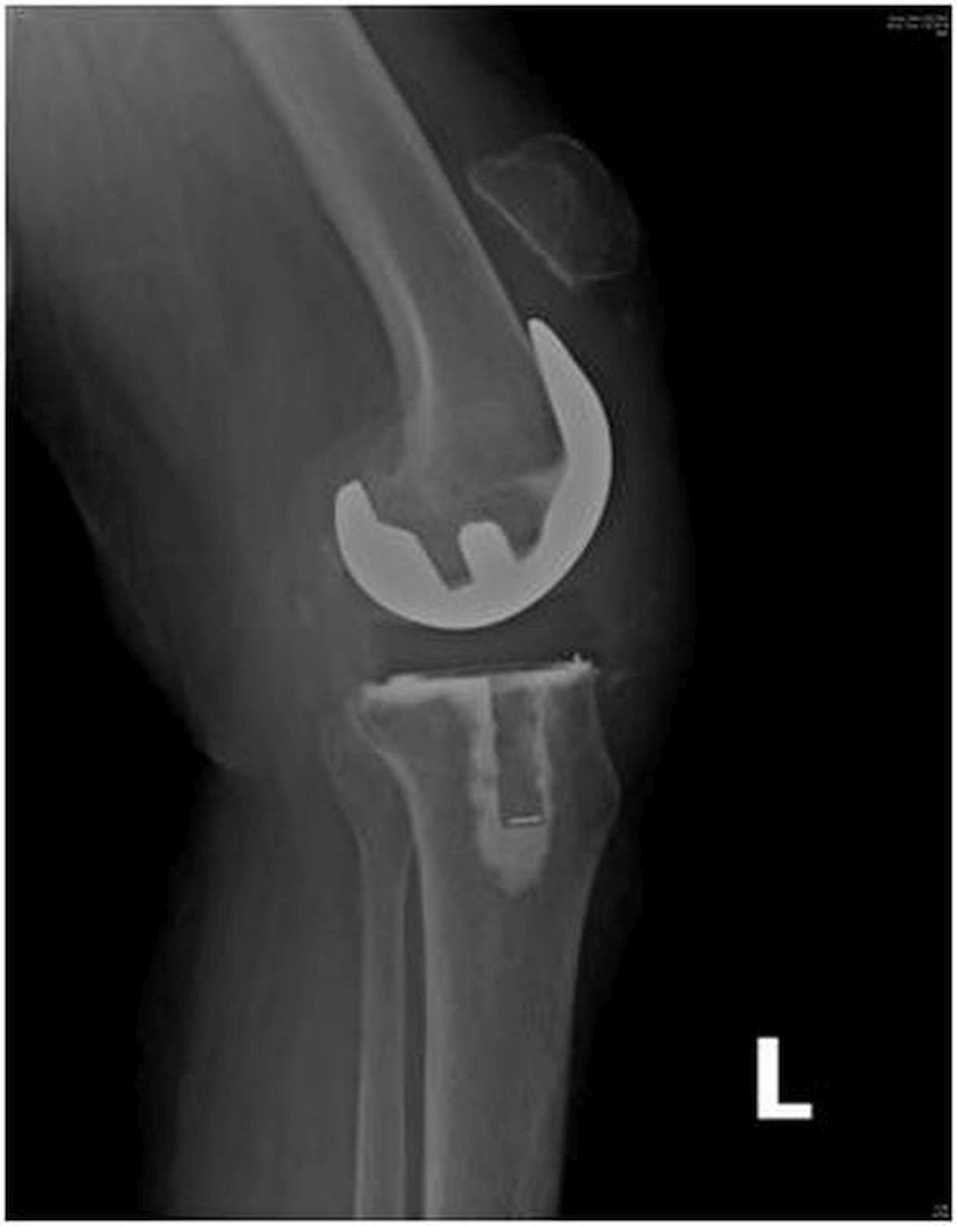
Lateral radiograph of a patient showing an infrapatellar tendon rupture and proximal migration of the left patella.

Out of 10 knees, five knees had a fractured patella, two sustained infrapatellar tendon ruptures (Figure 5), one developed a lateral patellar dislocation, and the remaining two sustained a rupture of the quadriceps muscle in the suprapatellar region. Seven out of 10 knees were operated upon while three were treated conservatively (Table 1).

**Table 1:** Table showing a series of nine cases (with 10 knees) of extensor mechanism rupture associated with total knee arthroplasty

S. No.	Age & Sex	Dx	Mechanism of Injury	Rupture Site	Treatment	Follow-up Duration	Extensor Lag	R.O.M.
1	72/F	R.A	Trauma due to fall	Infrapatellar	Reconstruction autologous semitendinosus graft.	6 months	45 deg.	0-100 deg
2	56/M	O.A	Trauma due to fall	Suprapatellar	Direct repair	9 months	10 deg.	0-120 deg
3	65/M	O.A	Trauma due to fall	Parapatellar	Lateral retinacular release	12 months	10 deg.	5-100 deg
4	65/M	O.A	Trauma due to fall	Transpatellar	Intra-osseus and retinacular repair.	12 months	20 deg.	10-90 deg
5	68/F	O.A	Trauma due to fall	Infrapatellar	Knee brace (patient refused surgery)	13 months	30 deg.	0-120 deg
6	32/F	R.A.	Forceful bending of knee	Transpatellar	Knee brace	16 months	10 deg.	0-45 deg
7	55/F	O.A	Intraop	Transpatellar	Knee brace	6 months	15 deg.	0-100 deg.
8	67/F	R.A.	Forceful bending of knee	Suprapatellar	Direct repair	8 months	10	0-110 deg
9	52/F	R.A.	Forceful bending of knee	Transpatellar	TBW	12 months	10	0-120 deg
10	64/F	OA	Forceful bending of knee	Transpatellar	TBW	14 months	10	0-100 deg

**Figure 6:**
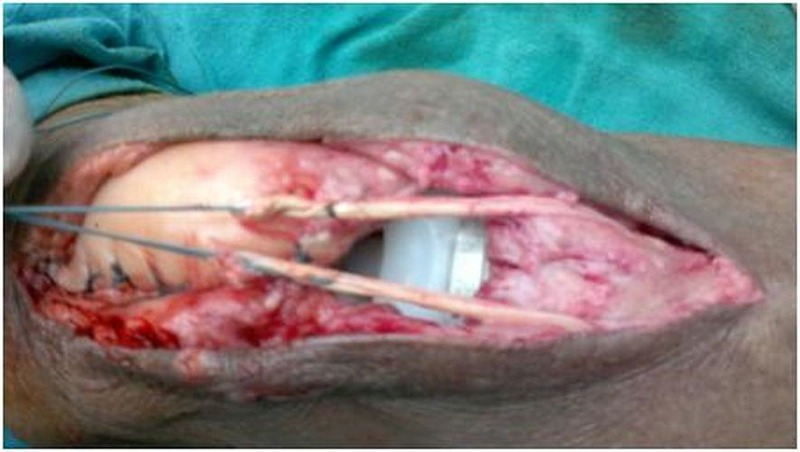
Intraoperative picture showing an infrapatellar tendon reconstruction using an ipsilateral hamstring (gracilis) tendon.

Three out of five patients with patellar fracture were treated by open reduction and internal fixation (ORIF) of the patellae with tension band wiring. Two out of five patients underwent a direct repair with an Ethibond #5 suture done for the rupture of the quadriceps mechanism. A lateral retinacular release was performed on one knee while reconstruction of the infrapatellar tendon was done on the other knee using the ipsilateral hamstring (gracilis) tendon (Figure 6). 

**Figure 7:**
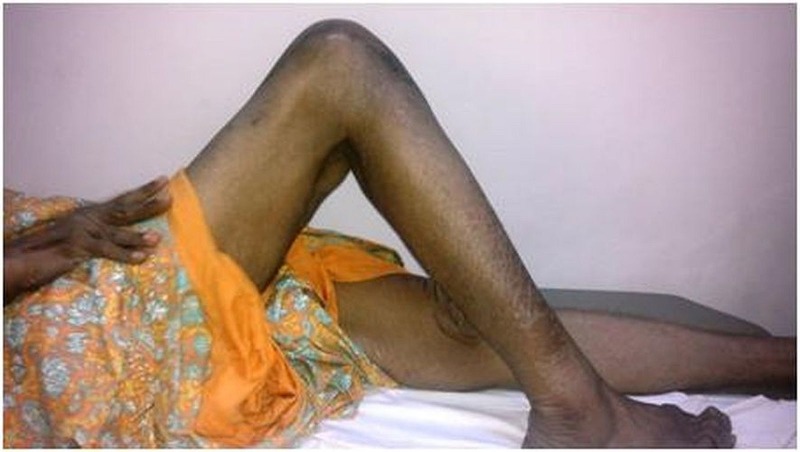
Clinical picture at six months follow-up, after infrapatellar tendon reconstruction showing a ROM with up to 100 degrees flexion.

A drill hole of 4.5 mm was made through the tibia at the tibial tuberosity level as well as another hole made through the patella in the mediolateral direction. The harvested tendon was passed through these drill holes and sutured back to itself and further anchored to the surrounding soft tissues. An above-the-knee cast was applied for one month followed by physiotherapy exercises. At the six-month follow-up, there was no recurrence of the dislocation and the ROM was up to 100 degrees flexion, but with an extensor lag of 45 degrees (Figure 7).

All of these nine patients were followed up for six to 16 months (average: 10.57 months) and were found to have some degree of extensor lag, ranging from 10 to 45 degrees (average: 20 degrees). The patients with infrapatellar tendon ruptures had that maximum lag while the suprapatellar lesions produced the least lag (Table 1). The average ROM after treatment of the extensor mechanism was 94.5 degrees (range: 0-120 degrees). 

## Discussion

Disruption of the extensor mechanism after TKA may result in a) patellar fracture and b) rupture of either the quadriceps tendon or c) the patellar tendon (PT). Our experience of extensor mechanism disruption after primary TKA is similar to that which is reported in the literature. Extensor mechanism rupture following TKA is a rare (0.7-2.7%), albeit devastating, complication [3]. If untreated, it leads to the inability of the patient to ambulate without a significant brace support or ambulatory aids and an ultimate early failure of TKA.

Proper surgical management of extensor mechanism disruption remains controversial. The treatment options for the repair of PT include observation, direct primary repair, or reconstruction with a synthetic ligament, autograft, or allograft tissue [4]. All these treatment options have been reported with inconsistent results. A direct repair is often difficult or not possible, due to poor quality and an inadequate amount of tendon tissue [5]. Hence, PT reconstruction is required in these cases, using either autograft (e.g., hamstring tendons) or allografts. Allografts seem a simple solution to reconstruct the disrupted PT adequately. Options for allograft reconstruction include either an Achilles tendon bone block allograft or a whole extensor mechanism allograft. Surgical principals include rigid fixation of the host-allograft junction, coverage of the allograft tissue with as much autogenous tissue as possible, tensioning the graft in full extension, and avoid testing the repair. With proper surgical technique and rehabilitation, patients show acceptable functional outcome for this devastating complication [6].

Some stable and undisplaced fractures of the patella can be managed nonsurgically [7]. Displaced fractures of patella or tendon rupture often lead to poor results. Patellar fractures after TKA are not very common. The factors considered for treatment include a) integrity of the extensor mechanism, b) fixation status of the patellar implant, and c) quality of the bone. Stable fractures associated with a stable implant can be treated successfully with nonoperative means with minimal complications. When operative treatment is required, it is often associated with a high rate of complications and surgeries. Patellar clunk, soft-tissue adhesions, prosthetic wear or loosening, and osteonecrosis are the other possible complications [8].

Quadriceps tendon tears associated with TKA are more difficult to treat and associated with a poor prognosis than quadriceps tendon tears in the native knee. Tears of the quadriceps tendon following TKA are less frequent, and only a few reports have addressed this complication [9]. While a single traumatic episode may result in a quadriceps tendon tear, systemic diseases, such as rheumatoid arthritis, diabetes mellitus, chronic renal failure, obesity, and hyperthyroidism, may predispose an individual to this injury. Local factors, such as prior knee arthroplasty, arthrotomy, multiple steroid injections into the knee, poor patellar positioning, and lateral retinacular release at the time of the total knee arthroplasty, have been associated with tears of the quadriceps tendon after TKA. In patients with a TKA, however, the results after primary repair of a complete quadriceps tendon tear have been less predictable, and no recommendations for the treatment of a partial quadriceps tear following TKA have been proposed.

In a study of 11 patients who had a complete rupture of the quadriceps tendon, nine had an identifiable predisposing systemic or local factor that may have contributed to the tear.  Only four of these 11 patients ultimately rated the outcome as satisfactory. Four of the 10 patients had a re-rupture of the quadriceps tendon after operative repair. Two patients had a deep periprosthetic infection develop and the TKA was removed. One of those two patients later had an above-the-knee amputation to control the infection.

In a review of 281 TKAs over a six-year period that included patellar resurfacing, there were 28 (10%) complications related to extensor mechanism: three quadriceps tendon ruptures, five patellar fractures, four PT ruptures, 11 recurring patellar subluxations, four cases of patellar pain, and one mal-rotated patella. Patellar fractures occurred mainly in patients who had RA. Patients with patellar instability had abnormal valgus deformities of their knees preoperatively. These patients presented with subluxation problems at an average of four months after surgery. The quadriceps tendon tear after TKA occurs in about 0.1% cases. We agree that the outcomes are poor for patients who have a complete quadriceps tendon tear after TKA. Suprapatellar ruptures in the quadriceps muscle could be due to improper repair of the quadriceps muscle or tendon during closure and may lead to failure with trauma. These ruptures can be repaired directly and are associated with good results and minimal extensor lag.

Our choice of reconstruction is the hamstring tendon autograft, as they are readily available locally around the knee and are free for the patient [10]. The selection of allografts is compounded by many inherent problems, such as non-universal availability, high cost, the possibility of disease transmission, and doubtful incorporation. The patients who sustain a partial tear could be successfully treated non-operatively without many complications and have uniformly good outcomes. Amongst all types of ruptures, it has been found historically that the infrapatellar ruptures of the patellar tendon are associated with poor results and have maximum extensor lag postoperatively.

**Figure 8:**
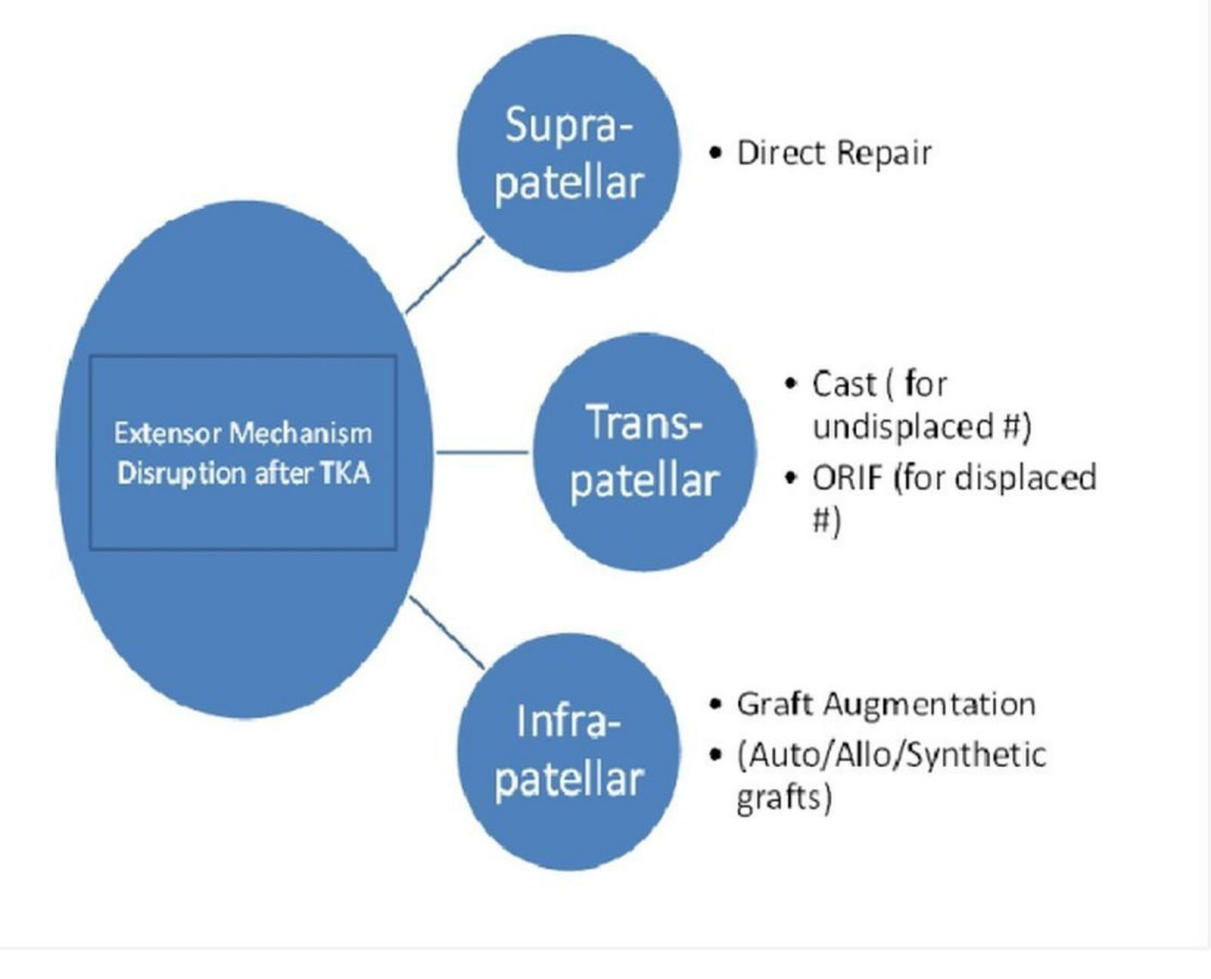
Algorithm for management of an extensor mechanism disruption after total knee arthroplasty

In TKA, most complications related to the extensor mechanism are reportedly caused by patellar maltracking or instability. Patellar maltracking may result from component malposition and limb malalignment, prosthetic design, improper patellar preparation, or soft-tissue imbalance. Patellofemoral instability commonly occurs from malrotation of the femoral or tibial components. Although plain (axial) radiographs may display the lateral subluxation of the patella, only computed tomography (CT) can quantify rotational malalignment of the components. Nonsurgical treatment is unsuccessful. A revision surgery is advised for major malposition of components. In the absence of component malposition, proximal soft tissue realignments (lateral patellar retinacular release) or tibial tubercle transfers have been used. Surgical repair or reconstruction of PT itself may risk rupture of the extensor mechanism. From our experience, we found that an injury in the first few weeks following an arthroplasty poses a maximum risk of extensor mechanism rupture. Intraoperative care must be taken to avoid an extensor mechanism injury like forceful eversion of the patella (using towel clips in a soft bone). We have provided an algorithm for management of extensor mechanism disruption after TKA (Figure 8).

Increased understanding of biomechanics, implant designs and alignment, rotation, and soft-tissue balance has resulted in a decrease in extensor mechanism-associated complications after TKA. Appropriate prosthetic selection and meticulous surgical technique remain the keys to avoiding unsatisfactory results and revision surgery.

## Conclusions

Extensor mechanism disruption as a result of patellar tendon, quadriceps tendon rupture, or patella fracture is an uncommon but devastating complication after total knee arthro­plasty. Various treatment options for repair of the extensor mechanism include observation, direct primary repair, direct primary repair with synthetic ligament or autogenous tissue augmentation, or reconstruction with allograft tissue. Recognition of the risk factors and prevention via meticulous surgical technique should be used in every patient undergoing TKA.
